# Unusual Instance of Mucosa-Associated Lymphoid Tissue (MALT) Lymphoma Confined to a Colonic Polyp

**DOI:** 10.7759/cureus.36791

**Published:** 2023-03-28

**Authors:** Amy Kiamos, Reeba Omman, JR Quan

**Affiliations:** 1 Internal Medicine, University of Florida College of Medicine, Jacksonville, USA; 2 Pathology, University of Florida College of Medicine, Jacksonville, USA; 3 Oncology, University of Florida College of Medicine, Jacksonville, USA

**Keywords:** marginal zone lymphoma (mzl), extranodal lymphoma of the colon, colon cancer and colon polyps, extranodal marginal zone malt lymphoma, extranodal malt lymphoma, mucosa-associated lymphoid tissue (malt) lymphoma

## Abstract

Extranodal marginal zone lymphoma (EMZL) of mucosa-associated lymphoid tissue (MALT) commonly affects the gastrointestinal (GI) tract but rarely occurs within the colon. Colonic EMZL is a rare diagnosis accounting for 2.5% of EMZL and less than 0.5% of colon cancers. We present a unique case of asymptomatic colonic EMZL diagnosed on a routine surveillance colonoscopy. The lymphoma was confined to a single colonic polyp presenting endoscopically as a sessile polypoid lesion at the recto-sigmoid junction. The patient was successfully treated with polypectomy with no recurrence of the disease.

## Introduction

Extranodal marginal zone lymphoma (EMZL) of mucosa-associated lymphoid tissue (MALT), also known as MALT lymphoma, accounts for approximately 7%-8% of newly diagnosed non-Hodgkin B-cell lymphomas [1]. EMZL may arise in several organs in the body; however, the gastrointestinal (GI) tract is the most frequently affected area encompassing 66% of cases [2]. The most common GI location is the stomach, accounting for 35% of total cases, followed by the small intestine [3]. While EMZL has a predilection for the GI tract, colonic EMZL is a rare entity. Colonic EMZL accounts for 2.5% of MALT lymphomas and less than 0.5% of colon cancers [4]. We present a case of a 71-year-old male with EMZL confined to a colonic polyp that was diagnosed on a routine surveillance colonoscopy. He was treated with polypectomy and has been cancer-free since resection.

## Case presentation

A 71-year-old male with a past medical history of human immunodeficiency virus (HIV) on highly active antiretroviral therapy and treatment for hepatitis C and hypertension presented to a gastroenterology clinic for a routine screening colonoscopy. He underwent a prior colonoscopy with the removal of two benign polyps five years earlier. He denied fever, unintentional weight loss, night sweats, palpable lymphadenopathy, hematochezia, melena, rectal bleeding, constipation, or diarrhea. The patient's vital signs consisted of a temperature of 97.7°F, a pulse rate of 61 beats per minute, a blood pressure of 135/73 mmHg, a respiratory rate of 17 breaths per minute, and an oxygen saturation of 100% on room air. His physical examination was unremarkable.

**Figure 1:**
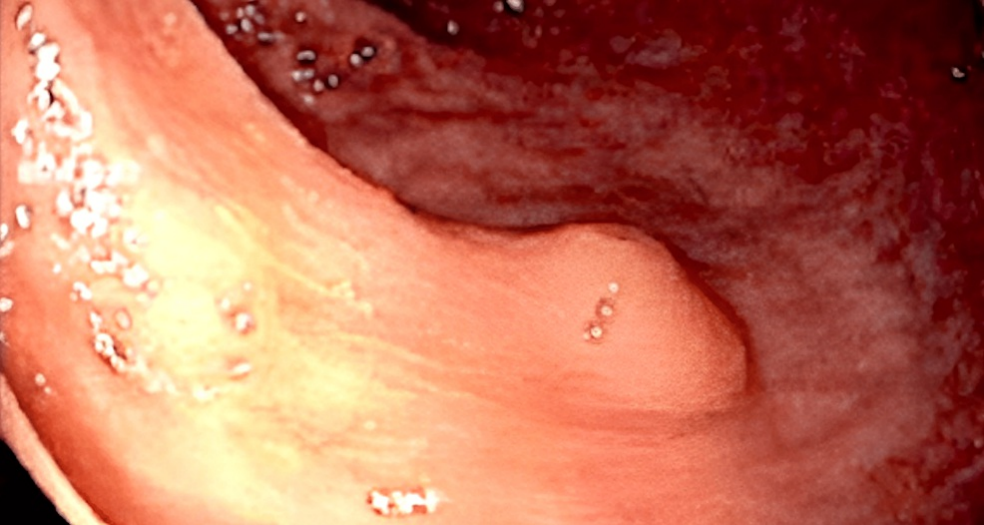
Endoscopic visualization of a 6 mm sessile recto-sigmoid polyp with normal-appearing colonic mucosa.

A colonoscopy examination revealed a 6 mm sessile polyp that was resected from the recto-sigmoid colon (Figure 1).

**Figure 2:**
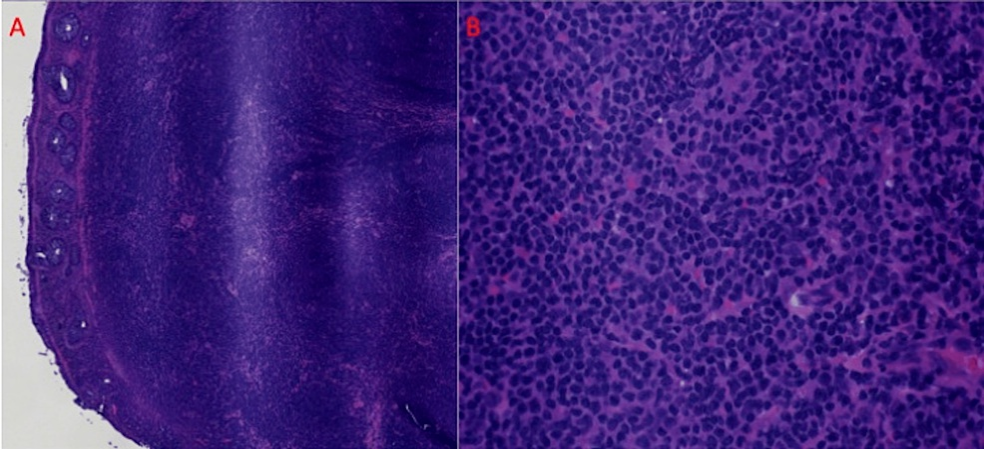
(A) Hematoxylin and eosin (H&E) section showing a low-power view of polyp with overlying colonic mucosa and underlying dense lymphoid infiltrate. (B) H&E section showing a high-power view of atypical small lymphocytes and plasma cells.

Surgical pathology of the polyp demonstrated a dense lymphoid infiltrate with atypical small-sized lymphocytes and plasma cells in addition to a few reactive germinal centers (Figure 2).

**Figure 3:**
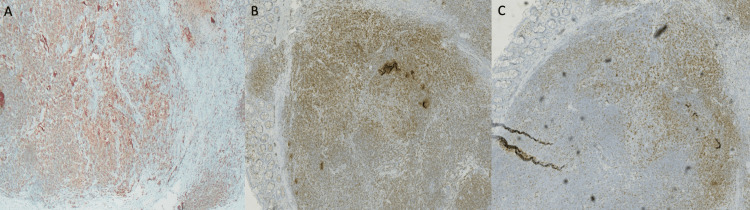
Immunohistochemical stain showing atypical lymphocytes expressing (A) CD20, (B) BCL-2, and (C) CD5 weakly. CD20, cluster of differentiation 20; BCL-2, B-cell lymphoma antigen 2; CD5, cluster of differentiation 5

Immunohistochemical (IHC) stains of the atypical lymphocytes were positive for cluster of differentiation (CD) 20, CD5 (weakly), and B-cell lymphoma antigen 2** **(BCL-2). IHC testing was negative for B-cell lymphoma antigen 1 (BCL-1), CD21, and cyclin D1 (Figure 3).

Additional testing showed a Ki-67 proliferation index of 10%. Overall pathology findings were consistent with low grade B-cell lymphoma, favorably marginal zone non-Hodgkin lymphoma.

He established close follow-up with oncology and underwent staging evaluation. His complete blood count and complete metabolic panel were within normal limits. Computed tomography (CT) of the neck, chest, abdomen, and pelvis was negative for pathologically enlarged lymph nodes. Bone marrow biopsy demonstrated no evidence of involvement by lymphoma. Given these results, no treatment was recommended. The patient was encouraged to have periodic colonoscopy examinations for further polyp surveillance every five years. He remains with no clinical evidence of the disease two years following diagnosis.

## Discussion

Marginal zone lymphomas (MZLs) are a collection of slow-growing non-Hodgkin B-cell lymphomas; there are three subtypes: extranodal MZL (also known as MALT lymphoma), splenic MZL, and nodal MZL [5]. MZL accounts for 7%-8% of non-Hodgkin B-cell lymphomas [1]. EMZL most frequently arises in the GI tract (66% of total cases) but may also be found less commonly in the lungs, skin, salivary glands, ocular adnexa, breasts, and thyroid glands [2,3]. Within the GI tract, the stomach is the most commonly affected site accounting for 60%-75% of GI cases, followed by the small intestine, rectum, cecum, and colon [6]. MALT lymphoma of the colon is a rare diagnosis accounting for only 2.5% of MALT lymphomas [4].

Colonic EMZL has a mean age diagnosis of approximately 60 years. Some studies show no sex preference, while others demonstrate a slightly higher incidence in females compared to males [1,3]. The most common presentation of patients with colonic EMZL is GI bleeding ranging from fecal occult blood to massive hemorrhage [4,7-9]. It can also present with nonspecific symptoms such as abdominal pain and rarely can cause intestinal perforation, intussusception, or obstruction [4,7,8]. Systemic B symptoms (fever, weight loss, and night sweats) are uncommon as EMZL is indolent and usually localized [1]. Patients may also present entirely asymptomatic, such as our patient, further highlighting the importance of routine screening colonoscopy.

The EMZL of mucosa-associated lymphoid tissue was first described by pathologists Isaacson and Wright in 1983 [2,5]. Histologically, it is defined by neoplastic cellular heterogeneity with centrocyte-like cells, monocytoid B-cells, small lymphocytes, and plasma cells [2,5]. There are usually reactive follicles present with heterogeneous neoplastic cells in the marginal zone and interfollicular region [2,5]. Immunophenotypic features of the neoplastic cells help to confirm the diagnosis. The cells express B-cell markers such as CD19, CD20, CD22, CD79a, CD79b, and BCL-2 and are negative for CD5, CD10, CD3, and CD23 [2,3]. Rare cases of MALT lymphoma can have CD5 reactivity with only less than 5% of cases reported, which further highlights the unique immunophenotype of our case [10].

The endoscopic appearance of colonic MALT lymphoma has varied in the few cases reported. Presentation can range from a single sessile polypoid lesion (most common) to multiple intestinal polyps [1,7,11]. The ulceration of the colonic mucosa is frequently noted; however, there have been diagnoses made from simple mucosal discoloration or normal-appearing mucosa [12]. Within the colon, tumor growth occurred more often in the rectum and cecum, followed by lesions in the ascending colon; however, the sigmoid colon is rarely involved [8,13].

Due to the rarity of cases, there are no guidelines for the standardized treatment of colonic EMZL. Currently, treatment is guided by the extension and stage of the disease. Low-grade locally limited colonic EMZL is treated with endoscopic or surgical local excision, while EMZL with the extension of the disease to other organs has been treated with surgery in addition to radiotherapy, chemotherapy, and sometimes rituximab [1,7,8,14]. There have been few case reports that have demonstrated regression of colonic EMZL with *Helicobacter pylori* treatment; however, *H. pylori* infection has not been well-established as a risk in comparison to gastric EMZL [15,16]. A well-established risk factor is immunosuppression, such as human immunodeficiency virus (HIV) [17]. Most studies have shown no improvement with *H. pylori* treatment; therefore, testing for *H. pylori* and antibiotics for eradication is not recommended [18]. In this case, our patient underwent polypectomy with no signs of recurrence two years later.

## Conclusions

This case highlights a rare presentation of an asymptomatic patient diagnosed with colonic EMZL on routine surveillance colonoscopy. The lymphoma was confined to a single colonic polyp and was successfully treated with polypectomy. EMZL is rarely found in the colon. Due to the limited cases reported, there are no standard guidelines for treatment. Although colonic EMZL is infrequently described, EMZL should be considered a differential diagnosis for colonic polyps.
